# Welcome to the future: challenges and opportunities discussed in the Vision 2048 Task Force Open Forums 2021–2023

**DOI:** 10.5195/jmla.2024.1970

**Published:** 2024-07-01

**Authors:** Charlotte M. Beyer, Janet Crum, Heidi Sue Adams, Roy E. Brown, Helen-Ann Brown Epstein, Jordan Dias Correia, Krystal Madkins, Matthew Nicholas Noe, Mary Joan (M.J.) Tooey

**Affiliations:** 1 charlotte.beyer@rosalindfranklin.edu, Associate Vice President of the Boxer Library, Rosalind Franklin University of Medicine and Science, North Chicago, IL; 2 janetcrum@mail.fresnostate.edu, Dean of the Library, Fresno State University, Fresno, CA; 3 hadams@logan.org, Lead Medical Librarian, Logan Health Medical Center, Kalispell, MT; 4 rebrown2@vcu.edu, Research and Education Librarian, Tompkins McCaw Library, Virginia Commonwealth University, Richmond, VA; 5 hepstein@virtua.org, Informationist, Health Sciences Library, Virtua Health, Mt. Laurel, NJ; 6 jcorreia2@nymc.edu, Research Services Librarian, Philip Capozzi, M.D. Library, New York Medical College, Valhalla, NY; 7 krystal.madkins@northwestern.edu, Education & Curriculum Librarian, Galter Health Sciences Library, Northwestern University, Chicago, IL; 8 matthew_noe@hms.harvard.edu, Lead Collection and Knowledge Management Librarian, Countway Library, Harvard Medical School, Boston, MA; 9 mjtooey@hshsl.umaryland.edu, Retired Dean and Associate Vice Provost & Library Faculty Emerita, University of Maryland, Baltimore; Baltimore, MD

## INTRODUCTION

What will librarianship look like in the future? This question is on the minds of health sciences librarians as our landscape of professional practice continually changes. As the Medical Library Association approaches its 125th anniversary, how can we build upon our rich past to build a better, more inclusive future? In 2020, the MLA Board of Directors transformed this question into a goal in the Association's strategic plan and appointed four task forces to address it. One of those, the Vision 2048 Task Force, was charged with engaging the MLA membership in a discussion of future profession in 2048. This article documents the work of the task force, the challenges that emerged in the various discussions, and opportunities for the association to consider as we move forward toward 2048 and beyond.

## THE VISION 2048 TASK FORCE

### Charge

The Vision 2048 Task Force is one of four task forces associated with MLA's Building a Better Future strategic goal. Its charge was, “to envision the future (25 years) of the profession with community-driven activities and ideation that reflects the richness of MLA communities” [1]. The task force met this goal by collaborating with MLA Caucuses, MLA editorial boards, and MLA headquarters to ensure broad participation in activities and discussions that engage perspectives from across the association.

### Membership

The goal of the task force was to have a diverse membership which reflected various perspectives within MLA. Members of the task force included one liaison from the Board of Directors to facilitate communication between the task force and MLA leadership, one member of MLA staff for project management purposes, a handful of volunteers who answered an open call through community council, and additional members with specific areas of expertise. Like the other 125th taskforces this one was appointed by the MLA president Lisa Traditi. The task force met monthly from June 2021 until early 2024.

Early on, the task force chair recognized that library school students were missing in the process. After a call for volunteers to include library school student perspectives, six students expressed interest to potentially serve on the task force. As the chair did not want the task force to grow too large, but still wanted students to be included in this initiative, the Vision 2048 Student Workgroup was formed. The goal of this workgroup was to serve as a focus group of library school students on library student specific challenges, issues, and concerns. To ensure the perspectives of this work group were incorporated into the main task force discussions and initiatives, two members of the workgroup were selected to serve on the main task force as liaisons. The group was facilitated by M.J. Tooey who was also a member of the main task force. All together the Vision 2048 Task Force and Vision 2048 Student Work Group consisted of 19 members: 15 librarians and 6 students.

### Meetings & Initiatives

The task force met monthly beginning in June 2021 to identify ways to engage MLA members. Ideas included doing an environmental scan, surveying MLA members and non-members, and conducting focus groups. After assessing the bandwidth of the task force members, the task force decided to host a series of town halls to get feedback from large groups of people at once. Most of the task force work then became dedicated to planning and hosting these town halls and evaluating information gained from them. This article summarizes the themes identified in these discussions and our recommendations to MLA based on those themes.

## THE VISION 2048 TOWN HALLS

Between 2021 and 2023, the Task Force hosted four town hall discussions, three with MLA members and one with library school students. The town halls were facilitated by members of the task force and current or past chairs of MLA caucuses. In keeping with MLA's strategic plan, MLA President Shannon Jones charged the task force with weaving Diversity, Equity, and Inclusion (DEI) into all of the open forum topics.

### Town Halls for MLA Members

Two of the town halls for members were held online and one in person at MLA 2022 in New Orleans. Each of the online town halls had around 100 attendees and the in person had just above thirty participants. Each event opened with a brief introduction followed by breakout groups focused on topics selected by the Task Force. The discussions in the breakout groups were facilitated by members of the task force or current/former MLA caucus leaders that were selected by the task force chair. The caucus chairs were selected based on expertise in relation to the topic they would facilitate. These topics covered all aspects of health sciences librarianship:

- Leadership and Management
- Clinical Outreach/Hospital Librarianship
- Community Engagement & Outreach
- Curricular Involvement
- Technical Services
- Collection Management
- Data Management and Digital Curation
- Reference & Consultation
- Research & Publication
- Educating Health Care Professionals
- Educating Future Health Information Professionals
- Web and UX (User Experience) Design
- Future Roles
- Healthy Work Environments
- Professional Library Associations
- Miscellaneous

Attendees at the virtual forums could share their thoughts verbally or via Zoom chat. Each session concluded with breakout groups reporting the results of their discussions to all attendees. Immediately after each session, facilitators sent notes and highlights from their discussions to the task force chair. This information informed discussions at subsequent Task Force meetings. Attendees also had the option of emailing the task force chair to share additional feedback after the town halls concluded.

### Town Hall for Students

In November 2022, the student work group hosted a virtual town hall for library school students. The session was open to MLA members and non-members and advertised to the MLA New Members Caucus and via library school email lists. This session consisted of a single large-group discussion rather than breakout sessions with around 40 attendees which included current library school students, members of the task force, and experienced librarians who came to hear the perspective of the students in attendance. Although participants were invited to share their thoughts verbally, most participated via Zoom chat. The chat transcript served as notes for the session. Some students also sent additional comments to the task force chair after the session.

## TASK FORCE FINDINGS

### Overarching Theme: Building a Better Future Through Centering the Needs of the Communities We Serve

As the task force analyzed feedback from these open forums, several themes emerged as important to MLA members. The largest of these themes was the importance of centering the needs of the communities health information professionals serve and adapting professional practice as the needs of these communities change. The undercurrent of constant change was ever-present throughout forum conversations in a variety of ways. Examples include the need for training and professional development for library professionals to gain new skills/competencies, preparing library school students for future practice in health sciences librarianship, advocating for the value librarians provide in academic and clinical environments, and adapting to changes in scholarly publishing and collection development.

The demographics of our communities are also changing, accelerating the importance of DEI principles in all areas of library practice. To build a strong future, we must include and engage a variety of perspectives. In her 2018 presidential address as the first Black president of the Medical Library Association, Beverly Murphy called MLA members to action by stating,

Diversity drives excellence and makes us smarter, especially when we welcome it into our lives, our libraries, and our profession. And we are smart. The diversity of our staff and our organization is important and it's necessary to help us survive and thrive in this journey. The melding of many different minds and thoughts, activities, feelings, and interactions produces a plethora of healthy, productive experiences that we all can gain from if we remain open and flexible. [2]

The rest of this article will document the sub-themes discussed in the open forums through the lens of challenges and opportunities MLA should consider to ensure that the membership is equipped to thrive in the next 25 years and beyond.

### Challenges and Opportunities

#### Recruiting the Next Generation of Health Sciences Librarians

The task force sought feedback on recruiting future health sciences librarians from current MLA members and from current library school students and recent graduates. One of the ways this was done was through the student town hall held in November 2022 which was mentioned earlier in this article. Participants identified challenges that begin before library school and continue during library school and after graduation.

##### Before Library School

Low salaries and limited budgets are a challenge for recruiting into the library profession generally, including health sciences librarianship. Many other professions that require graduate degrees pay more than librarianship. Further, many health sciences library positions are with nonprofit or public sector employers, which generally pay less than private sector employers.

Health sciences librarianship also suffers from a lack of visibility; most non-librarians are unaware that our profession exists. Many suggestions from MLA members are aimed at addressing this lack of visibility. Members suggested the need for programs aimed at K-12 and undergraduate students to expose them to medical librarianship. Specific suggestions included developing pipeline programs, creating tool kits for high school and undergraduate curricula, including medical librarianship in STEM outreach programs, and creating programs similar to the First Look Program from AMIA to expose undergraduates to our field. Some participants also mentioned creating programs to recruit non-librarian professionals (e.g., practicing health professionals) into health sciences librarianship.

##### During Library School

The overall lack of consistent inclusion of specializations and health sciences librarianship training within graduate library education is among the greatest challenges facing health sciences libraries, health sciences librarianship, and the Medical Library Association (MLA.) These challenges were mentioned numerous times both in the general town halls and in the town hall for students.

Many Master of Library and Information Studies students are unaware of health sciences librarianship, some students think one must have an academic background in the health sciences to be a health sciences librarian. Many programs lack robust health sciences tracks or other means of exposing students to our profession. There is a general lack of knowledge regarding which library and information schools offer coursework, let alone tracks, in health sciences librarianship. While many schools promote courses or a specialization in health sciences librarianship, classes may be offered irregularly or qualified faculty to teach the courses. The MLA Professional Recruitment and Retention Committee (PRRC) maintains a list of library schools offering health sciences courses but does not verify whether or how often the courses are actually offered.

Basic core classes and competencies in health sciences librarianship are also not defined. Introductory courses defining and explaining the health sciences library environments—academic, hospital, special—are not provided nor are courses regarding useful skill sets and practical needs. MLA is developing a set of core competencies and a curriculum. It is unclear how this curriculum will be shared with library schools or if individuals will need to pursue these competencies outside of library education.

Many town hall participants agreed that the best learning experiences come through practicums, internships, or fellowships, but they may be out of reach for students with limited financial means or the mobility to travel to remote locations for limited experiences. Most of these opportunities offer course credit in lieu of salary, and course credit does not pay for living expenses. Some participants mentioned that they need to work to survive while enrolled in graduate school, so unpaid opportunities would not be an option. Some internships do offer financial support but often require students to relocate, adding to the burden of travel costs, potentially additional rent, and increased cost of living. Online experiences are available, especially since the pandemic, and are particularly valuable to part-time or working students. Online opportunities are more effective in some areas and may not offer direct interactions with other library staff or users. Shadowing library staff is another learning option for library school students depending on geographic proximity and opportunity.

Not everything can be learned in library school, so how do we prepare students for diverse opportunities and changing environments? In the general town halls, experienced librarians admitted frustration with library schools and indicated that most training occurs once a new librarian is hired. This necessitates investment in time and training but allows the new librarian to acclimate to the work environment and its needs. Professional associations such as MLA can help to “skill up” librarians with programs such as the Research Training Institute (RTI), multi-level continuing education courses (e.g., the systematic review specialization courses), and exposure to new and emerging specializations such as data science/visualization, health informatics, working with the research enterprise, or artificial intelligence. Many of these programs are not free and must be funded by either the individual librarian or their employer.

Recommendations from town hall participants fell into two categories. One is building relationships with library schools and strengthening health sciences curricula and learning opportunities within them. The second is building a strong student or new professional group within MLA. Both recommendations require effort and support from MLA.

Participants recommended that MLA build relationships with library schools and the Association for Library and Information Science Education (ALISE) to increase exposure to health sciences librarianship and support robust educational opportunities in the discipline. Participants suggested MLA should:

- Verify that health sciences courses advertised by library schools are robust and truly exist. MLA could maintain a clearinghouse of these programs and promote them, similar to a seal of approval.
- Host a summit for library schools who support health sciences librarianship education to discuss issues and needs. This would also be an excellent opportunity to introduce the new MLA curriculum and competencies and build partnerships. There may also be an opportunity for MLA to license this curriculum to library schools.

Ideas for a curriculum supporting health sciences librarianship include a two-part course on the health sciences library environment. Part one would take a deep dive into the types of health sciences libraries including academic, hospital, consumer health, federal, or pharmaceutical. The pivotal role of the National Library of Medicine (NLM) would be included, providing a segue into the second part. The second part would focus on practical knowledge and skills such as Medical Subject Headings, data structures, systematic reviews, cultural competency, and Health Insurance Portability and Accountability Act/privacy regulations. Participants also recommended a library marketing, planning and advocacy course that included promoting expertise, relevance and impact along with strategic planning and alignment. Other ideas included user interactions/customer service, teaching/training, information/knowledge management, writing and communications (including National Institutes of Health biosketches and grants), and soft skills such as collaboration and interpersonal communication to support effective team collaboration and integration into institutional programs. While many of these topics are general and essential in all types of libraries, frequently there are nuances in the health sciences environment that need to be addressed. These could also become continuing education credits or special webinars of interest to MLA members and others. Below are a few ways the Student Town Hall participants suggested MLA could do to support students:

- Maintain a clearinghouse of practicum, fellowship, or internship opportunities enabling members to post options and students to find them.
- Offer reduced student rates for continuing education and student scholarships to attend the annual meeting.
- Establish a student caucus and promote it to library schools along with other benefits for students.
- Offer course(s) in library school curricula, including the Research Training Institute.
- Develop an MLA course designed to help students secure their first professional position. It could include resume writing, interviewing, a panel of new librarians, and a panel of potential employers sharing what they look for in candidates.
- Co-host events with library schools.
- Encourage health sciences libraries to offer paid internships and advertise positions—with salary information—at library schools.

One discussion point that seemed to resonate in all the town halls was whether professional associations were still relevant. In libraries as well as in library schools, there are so many options for learning and professional engagement both connected and unconnected to professional associations. One other challenge reported by experienced health sciences librarians in the discussion groups was a gap in necessary skills in recent graduates who apply for librarian roles. For example, if applicants are not familiar with resources heavily used in the clinical setting, they may struggle in a hospital/clinical librarian role. Since MLA specializes in health sciences librarianship, it could serve as a place to help librarians develop the competencies needed to be successful in the health sciences, benefiting both new librarians and institutions hiring them. While many of the recommendations for graduate library education suggest and even require forceful commitment from MLA, devoting effort to the next generation of health sciences librarians and ensuring new librarians choose MLA as their professional home might be worth it.

##### After Library School—Entry into the Profession

Town hall participants also made suggestions for recruiting and supporting new librarians. Challenges for new librarians that could be addressed by MLA include the cost of membership, Academy of Health Information Professionals (AHIP) certification, continuing education courses, and conference attendance; the need for networking and mentoring opportunities; and the need for practical training focused on professional competencies such as the MLA Competencies for Lifelong Learning and Professional Success [3]. Training from MLA could also cover more general career competencies such as interviewing, negotiating job offers, avoiding toxic work environments, avoiding apathy and burnout, emotional intelligence, navigating hierarchies, critical thinking, communication skills, professional ethics, medical terminology, functioning as a solo librarian, orientation to higher education, demonstrating impact, and dismantling white supremacy in libraries, universities, and hospitals. Challenges that could be addressed by practicing health sciences librarians include creating job descriptions with language that is welcoming to new graduates, reconsidering experience requirements, assessing institutional procedures such as requiring employees to front expenses for professional development and wait to be reimbursed, and creating pathways for librarians in other areas of the profession to move into health sciences librarianship.

Members cited several issues related to DEI that impact the recruitment of health sciences librarians. These included: the need to make DEI information unified and findable on organization websites; barriers to accessibility created by outdated infrastructure and confusing policies and procedures; the need for affinity groups and mentoring based around shared identities increase belonging; the lack of DEI infrastructure for recruitment in hospital libraries; the need for baseline demographic data for LIS programs; and the ability for health sciences librarians to express their identities and bring their full, authentic selves to work.

### Changes In How People Find and Use Information

During the townhall discussions, members noted that changes in sociocultural, political, and technological landscapes continue to impact the way people find and use information. These changes are already being felt in library practice and members shared both challenges and opportunities with the task force.

#### Library Collection Composition

While the last two decades have seen the normalization of electronic resources, with scholarly journals and databases dominating the collection budgets of health sciences libraries, the next two decades are primed to see additional changes. Facilitating access to media – from video content to digital education tools like UWorld for medical school board preparation – continues to pose challenges for libraries as these tools strain budgets and the technical capabilities of our institutions.

Though physical collections shrank throughout the 2000s and 2010s, members reported increased scrutiny of physical space in light of changes to work and school life as a result of the COVID-19 pandemic. Libraries are being increasingly pressured to reduce physical collection (and even staff) footprints in favor of other uses, such as study space or for initiatives outside of the library. At the same time, there is increased patron use of mobile technology to access scholarly resources, which poses accessibility and usability challenges that library staff are often ill-trained or, in the case of vendor platforms, unable to address.

#### Purchasing and Licensing of Resources

Town hall attendees also discussed how libraries purchase materials. In the last three decades, library purchasing has shifted from one-time purchases of physical materials to subscriptions to electronic resources. As a result, libraries no longer own most of their collections, which in turn creates a cascade of concerns that include the inability to guarantee access (now or in the future), limited budget flexibility, and increased difficulty in explaining the return on investment for library resources. Coupled with straining budgets at universities and hospitals, libraries are limited in their ability to cultivate a collection responsive to user needs. Participants noted that members are asking MLA and other professional organizations for help negotiating with vendors and developing policies and best practices for licensing, ownership, and long-term access to library collections.

Members expressed concern at the sheer volume of publications being generated and the expectation of having access to all of them. More than just a budgetary problem, this growth in scholarly publications intersects with continued reductions in staff time for collection management work, leading to questions of quality vs quantity in acquisition decisions. While MLA cannot directly create more positions or staff time, it could potentially help members by evaluating new resources and/or providing a framework for doing so.

#### Data and Artificial Intelligence (AI) Tools in Library Collections

Members also discussed, with mixed feelings, the role of the library in providing access to and preservation of various types of data including data sets. Some viewed it as an area where librarians are well suited to use their expertise to fill needed roles in this field, while others expressed concerns related to lack of training and institutional resources to adequately support management of these types of collections/services. It would be a strain to consider this an emerging topic, as it has been a focus of NLM for at least a decade, but it remains an area that health sciences librarians are likely to seek out training and ideation about from MLA. During the town halls members also mentioned AI as a potential area for exploration. As AI - generative and otherwise - continues to emerge, members will undoubtedly be facing a slew of new financial and ethical challenges with the expectations of home institutions and patrons. At the time of these town halls, this was largely a future concern, but as is evidenced at the time of publication, change in this space is happening faster than anticipated.

#### Changes in Needed Librarian Expertise

Members of the library team which includes both those with and without library degrees are taking on new roles requiring new expertise. Members anticipate the library taking on responsibility for additional parts of the scholarly communication and data management workflows, including developing repositories for and facilitating paid access to datasets where additional training opportunities are needed.

#### Factors that Impact the Use of Information

Two factors impacting the use of information were at the forefront of town hall discussions: information overload and information literacy. As research and practice continue to become more interdisciplinary, and with the aforementioned increases in scholarship being published, there are very real risks of overload for even the most focused of patrons. To address information overload, librarians will need to carefully curate resources and make explicit connections between curricula, research, and library resources – something that MLA advocacy could help with. What this advocacy might look like will surely change over time, but two concrete examples include providing talking points for health sciences librarians to use when working with their parent institutions (variations for type of parent institution are vital) and working more closely with related professional associations to ensure staffed, funded health sciences libraries are a priority in accreditation and best practices.

Regarding information literacy, concerns are multifaceted. Librarians need to be better trained in teaching skills so that gaps can be addressed more effectively – and one-shot instruction sessions remain a thorn in the side of many instruction librarians. Members are noticing changes in the information-seeking behavior of those they work with. These changes are partly driven by deficits in information literacy, leading individuals to prematurely end searches or settle for information on a topic instead of striving to find the best or highest quality information to fulfill their needs. Moreover, individuals tend to seek out a singular answer to their questions rather than adopting a more comprehensive approach. These challenges are ones that librarians are well-equipped to tackle. Members also suggested that a more thorough adoption of the Framework for Information Literacy for Higher Education developed by the Association of College and Research Libraries (ACRL) may be a stepping stone on this path.

##### Impact of the Changing Political and Social Landscape on Information Use

There remain considerable political risks for health sciences librarians, particularly in the United States where some political activists are fighting to limit access to information on topics such as abortion, DEI/antiracism, and gender identity. Members noted the importance of advocacy from MLA and ALA to ensure access to information on topics around sexual orientation, gender identity, and sex education more broadly and that library workers remain protected. Climate change is another topic entrenched in political challenges that will become increasingly vital for health sciences librarians to address. From infrastructure stability to making connections between changing climates and health, information workers are going to play vital roles in the coming decades.

During the town halls, members noted the increased pressures librarianship is facing in this era of misinformation and disinformation and that there may be a need for us to take on the task of providing additional training in information literacy. This concern was at the forefront in 2023 when Michelle Kraft explored the long history of ‘fake news’ and the role of health sciences librarians in addressing it during her Janet Doe Lecture [4]. In addition to concern about bad actors in the information landscape, members also noted the emergence of generative AI and its potential to impact the information professions. In addition to the intellectual property, environmental, and labor concerns this technology brings with it, there is also great risk to the accuracy of information. These tools can generate false claims, citations, and worse. Members are seeking guidance and opportunities to better understand this technology, not only for its downfalls but for potential upsides as well including where librarians can partner with designers of AI tools to ensure accurate information is disseminated and shared.

### Retaining the Library Workforce

#### Work-Life Balance and Burnout

Town hall participants shared their thoughts on how health sciences libraries can best retain their staff, especially considering new work norms driven by the COVID-19 pandemic. Participants offered suggestions for library leaders, most focused on addressing the emotional well-being of librarians to improve retention.

In particular, members acknowledged that institutional policies, often rooted in traditional management philosophies prioritizing physical presence, may contribute to burnout and hurt retention efforts. Policies, procedures, and workplace norms must allow for all staff to be heard and included. To create this inclusive approach, participants suggested that leaders must develop a culture of open communication in which library staff feel safe and comfortable discussing their workloads, challenges, and concerns. Staff need multiple venues to communicate with leaders and managers as well as other library colleagues to address issues before they become serious problems.

To encourage work-life balance, participants suggested that medical libraries should implement flexible schedules, including compressed workweeks and/or flexible work hours, enabling librarians to meet both work and personal responsibilities. Libraries should also permit remote or hybrid work. Managers will need to balance the library's operational needs with the individual needs of their staff.

While flexible schedules focus on individual needs, managers also need to build cohesive teams. Participants agreed that team building is critical in reducing burnout. For medical librarians, collaboration within the discipline and with other disciplines is key. Establishing these connections can help mitigate loneliness and isolation, making individuals more resilient as they mutually support one another to address issues or meet the needs of the library. Enhancing team dynamics creates a positive work environment, reducing stress and burnout.

Additionally, participants agreed that managers should help librarians navigate this new work environment where physical presence is not always required. Remote/hybrid work arrangements can blur the boundaries between work and personal time, leading to increased stress and a heightened risk of burnout. Managers must collaborate with library staff in establishing norms for remote/hybrid work and teamwork. These norms should emphasize the importance of creating clear personal boundaries to maintain mental health, allowing time for personal pursuits and meaningful connections.

Participants shared that regardless of the steps taken, there will be times when librarians may feel overwhelmed, anxious, and unable to deal with their work, personal responsibilities, or both. It is key for managers to foster an environment where people understand that it is okay not to be okay and to ask for help. Leaders could offer opportunities for library staff to learn strategies to deal with stress, anxiety, setting boundaries, and other ways to cope or address the pressures of work while achieving work-life balance. Leaders should also ensure that staff are aware of employee assistance programs (EAPs) and know how to use them to get professional help when needed. MLA can help foster retention by providing courses, workshops, and other programming to help leaders and managers create healthy, supportive work environments. Potential topics include:

- building cohesive teams in a remote or hybrid environment
- improving emotional well-being
- promoting work-life balance
- clearly communicating role expectations
- conflict resolution

#### The Importance of Defining Roles & Boundaries

One other major topic discussed in relation to burnout was the ever-increasing duties and responsibilities assigned to librarians. Participants reported pressure from institutional leaders to prove the library's value. As a result, librarians may agree to take on new responsibilities and skills without letting go of other work, leading to overstretched and overwhelmed teams. Librarians from communities historically excluded from librarianship reported feeling burnout due to being volunteered without consent to serve on institutional committees and initiatives related to diversity. Many times, these initiatives are labor intensive and not related to their expertise, forcing them to do more work than their white colleagues. Town hall participants talked about the importance of library leaders being able to set boundaries and that MLA may be able to help by providing training and education in this area.

## PROFESSIONAL DEVELOPMENT IN THE FUTURE

**Figure 1:**
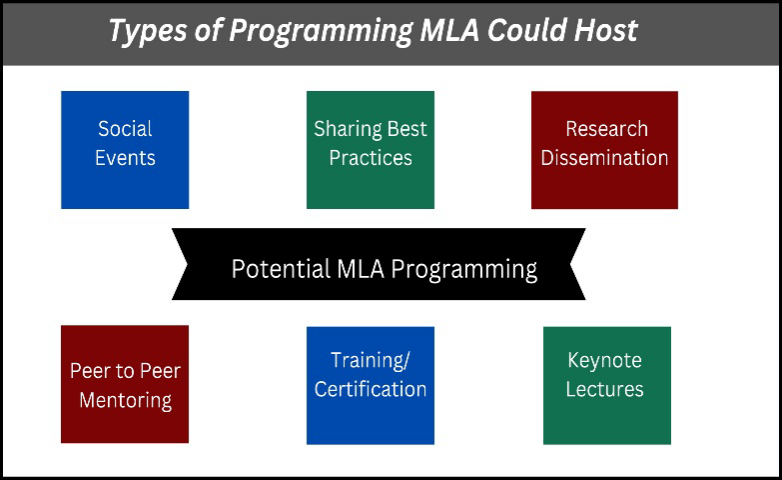
Programming MLA could host virtually and in person

For current and future librarians to adapt to changes noted in the town hall discussions, professional development must exist to not only support the present changes but to prepare for the future ones as well. When discussing professional development, two main themes emerged within these conversations which include the role of professional organizations such as the Medical Library Association as well as the role of individual professional development activities such as training and certification courses. Figure 1 presents types of programming MLA could do to support librarians’ development.

### Importance of Virtual Professional Development

Institutional funding is a key factor influencing the ways people engage in professional development. Participants in the member and student town halls were hesitant about spending substantial amounts of personal money on their professional development especially if there was not an immediate benefit to their career progression. With the pandemic, virtual options for professional development—webinars, certification courses, and virtual conferences—became an option for librarians to develop new expertise without travel costs. During the discussions, members encouraged MLA to explore and expand virtual training opportunities.

### The Importance of In-Person Professional Development

Despite a desire for continued options for virtual professional development options, members also indicated that in-person professional development is still valuable. The importance of the spontaneous human interaction that happens at an in-person conference or workshop was acknowledged to be difficult to replicate online. The collaboration through conversations and socialization happening in between sessions, at meals, or at social events can forge new relationships; these relationships can lead to collaborations and peer-to-peer support, which are invaluable to not only career development but also overall well-being that helps to minimize burnout. Participants talked about how just knowing someone else is going through the same thing can help with the mental load that comes with librarianship. This is especially true for librarians who are the sole person in their library from a historically excluded community; they may feel isolated with no one else representing the needs of their community. Having a place where they can come to physically connect helps them to find their professional home in a community of peers with similar life experiences. Members also noted that those in solo librarian roles also benefit from in-person connections.

Town hall participants noted the biggest challenge with these activities is the increasing costs associated with travel and registration. Those participants who had experience planning conferences shared how the costs of putting on an event are only increasing, which then drives up the cost of registration. Some suggestions for minimizing costs included encouraging vendors to fund more meeting grants, hosting events in smaller cities, and partnering with other organizations to have joint conferences. Members also suggested examining the frequency of large-scale meetings to alternate with other large library meetings such ACRL. In addition to examining the structure of conferences, MLA should prioritize content that allows for one-on-one engagement between members. The focus of conferences should be on relationship-building and problem-solving in real time. Presentations could be recorded and shared virtually so that in-person time is focused on human interaction.

### Importance of Advocacy & Partnerships

Participants in the town halls discussed the importance of partnerships outside of the library, including with other departments, university and clinical faculty, university and clinical administration, and community partners. How the library is perceived by these partners often has a direct impact on how the library functions within the institution and the impact it can have on members of their community.

As noted earlier, institutional policies have a large impact on the well-being of employees. Sometimes library workers, even library administrators, have very little say in the drafting and implementation of these policies and procedures. Town hall participants noted that MLA could provide a space for leaders to connect to one another to learn how to work with institutional partners to advocate for the library and include library professionals in decision making.

Participants also recommended that MLA leadership forge partnerships and collaborations with leaders at change-making organizations whose educational, research, or clinical missions align with MLA, its values, and the work of its members. Advocacy and partnerships were identified as vital components for hospital librarians as many hospital libraries close due to budget cuts.

### Role of Hospital Librarians in Clinical Care

During the town hall discussions, concerns from hospital librarians were woven throughout various discussions from advocacy to collaborating with academic librarians in educating the next generation of health care professionals. Members mentioned that not all hospital librarians work in an academic healthcare setting. While there is much overlap between academic and hospital librarianship, there are some differences to consider. Most academic settings have multiple librarians but there are numerous non-teaching hospitals who may employ only one librarian to provide library services, in many cases for multiple clinical locations. Participants discussed the need for increasing programs leveraging the interdisciplinary and multidisciplinary opportunities of hospital librarianship. MLA would do well to offer programs supporting this smaller membership subset. While hospital librarians want to volunteer for a variety of MLA roles and programs, hospitals often limit access to websites and do not provide institutional funding for activities not directly related to one's employment. These librarians are eager to secure funds for continuing education and attending local, regional, and national meetings so they can network with experts and colleagues and apply new knowledge to library programs and services. Hospital librarians feel pressure to demonstrate their value to hospital programs and services in education, patient care, and research.

Participants suggested that MLA continue expanding virtual meeting options, support technical accessibility, understand the trends in hospital financial structures and their impact on hospital librarians and their respective service areas, consider removing silos between academic and hospital librarians, and engage all types of medical librarians in association activities.

## CONCLUSION

Through these town halls, the Vision 2048 task force was able to engage with the membership to identify future challenges and opportunities for MLA. Through the conversations at the town halls and task force meetings, it was evident that MLA has a passionate and engaged membership invested in the future of health sciences librarianship. Together, members and association leadership can use the challenges and opportunities identified in town hall discussions to inspire future conversations and initiatives that support health sciences librarians and the communities we serve.
